# Acoustic signal perception in a noisy habitat: lessons from synchronising insects

**DOI:** 10.1007/s00359-012-0718-1

**Published:** 2012-03-17

**Authors:** M. Hartbauer, M. E. Siegert, I. Fertschai, H. Römer

**Affiliations:** Institute of Zoology, Karl-Franzens University Graz, Graz, Austria

**Keywords:** Acoustic communication, Background noise, Chorus synchrony, Signal detection, Entrainment

## Abstract

Acoustically communicating animals often have to cope with ambient noise that has the potential to interfere with the perception of conspecific signals. Here we use the synchronous display of mating signals in males of the tropical katydid *Mecopoda elongata* in order to assess the influence of nocturnal rainforest noise on signal perception. Loud background noise may disturb chorus synchrony either by masking the signals of males or by interaction of noisy events with the song oscillator. Phase-locked synchrony of males was studied under various signal-to-noise ratios (SNRs) using either native noise or the audio component of noise (<9 kHz). Synchronous entrainment was lost at a SNR of −3 dB when native noise was used, whereas with the audio component still 50 % of chirp periods matched the pacer period at a SNR of −7 dB. Since the chirp period of solo singing males remained almost unaffected by noise, our results suggest that masking interference limits chorus synchrony by rendering conspecific signals ambiguous. Further, entrainment with periodic artificial signals indicates that synchrony is achieved by ignoring heterospecific signals and attending to a conspecific signal period. Additionally, the encoding of conspecific chirps was studied in an auditory neuron under the same background noise regimes.

## Introduction

In real-world environments with many simultaneously active acoustic signallers, background noise has the potential to interfere with the perception of conspecific signals as soon as there is considerable overlap in the frequency content and if its intensity exceeds a critical threshold (Scharf 1970; Brumm and Slabbekoorn 2005). Therefore, ambient noise challenges the ability of receivers to detect conspecific signals in the presence of noise and discriminate these signals from those of heterospecifics. The task becomes even more difficult when receivers need to distinguish among different variants of conspecific signals in the context of mate choice (Andersson 1994; Gerhardt and Huber 2002; but see Einhäupl et al. 2011).

In nocturnal rainforests with a high number of different signallers active at the same time, the ambient noise can amount to 60–65 dB SPL (Lang et al. 2005). Mainly frogs and crickets contribute to sonic background noise (Riede 1997; Narins 1982; Ellinger and Hödl 2003), whereas various katydid species extend this noise to the high audio range and to ultrasound (Heller 1989; Morris et al. 1994; Balakrishnan 2005; Lang et al. 2005). In this complex acoustic situation, the possibility for receiver errors increases, and applications of game theory (Johnstone 1994, 1998; Johnstone and Earn 1999) as well as signal detection theory (Wiley 1994, 2000) highlight the importance of errors as a result of noise for the evolution of communication systems. This shaped the evolution of acoustic signal design and auditory systems to maintain effective communication in a noisy habitat (Richardson and Lengagne 2010). As a result of these adaptations, we should expect some tolerance against influences of acoustic background in acoustic communication of animals. For example, according to the “matched filter hypothesis” (Capranica and Moffat 1983; Gerhardt and Schwarz 2001) receivers gain an advantage from being tuned to the carrier frequency of conspecific signals, which is especially important in the presence of ambient noise. Recently, evidence for this hypothesis was provided in a tropical cricket community in which receivers evolved rather sharply tuned frequency filters with a best frequency tuned to the conspecific carrier frequency of calling songs (Schmidt et al. 2011). This improves the signal-to-noise ratio and therefore serves a similar function as the basilar papilla tuning in cricket frogs (Wilczynski et al. 1992; Witte et al. 2005).

The calling songs of katydids, however, are usually broadband signals with a frequency spectrum that extends far into the ultrasonic range; some include only ultrasonic frequencies, in single cases of tropical species up to more than 100 kHz (Heller 1989; Morris et al. 1994). Correspondingly, their ears and identified interneurons of the afferent auditory pathway are usually broadly tuned, which renders hearing in multi-species choruses especially vulnerable to masking. Katydids have to rely on the temporal pattern of advertisement signals for species recognition (Schul 1998), but this pattern may be severely degraded by heterospecific signals overlapping in time. Thus, even the competition with only one heterospecific signaller can have dramatic effects for signal perception and intraspecific communication (Greenfield 1988; Römer et al. 1989).

A number of different mechanisms have been reported both for the sender and receiver side in acoustic communication systems, which facilitate signal detection and discrimination in a noisy world (Klump 1996; Gerhardt and Huber 2002; Römer et al. 2002; Brumm and Slabbekoorn 2005; Ronacher et al. 2008). However, although katydids contribute significantly to the high audio and ultrasonic noise in different habitats, the knowledge about the influence of habitat noise on intraspecific acoustic communication and predator detection in this taxon is still in its infancy. As an exception a recent neurophysiological study revealed a remarkable neuronal representation of repetitive, bat-like echolocation signals typically for aerial hunting bats in katydid receivers, despite high levels of nocturnal rainforest noise (Hartbauer et al. 2010). Comparable studies for conspecific signalling are missing. In this study, we therefore investigated the influence of rainforest noise in an acoustically communicating insect and take advantage of the specific mechanism by which males of this species interact. When singing in isolation, males of the tropical katydid *Mecopoda elongata* produce broadband chirps (range from 2 to 95 kHz) very regularly in intervals of about 2 s. In an interaction with other males they synchronise their chirps with those of others, which results in a high degree of signal overlap (Sismondo 1990; Hartbauer et al. 2005). In this species, chorus synchrony is established by a phase change of the song oscillator after perception of signals that are out of phase with the production of chirps of the focal male. This mechanism of establishing chorus synchrony renders the underlying song oscillators vulnerable to the influence of heterospecific signals. In this system, rainforest noise may affect chorus synchrony in two different ways: (1) Via the effect of masking the chirp of neighbouring males, so that phase-locking to these chirps and thus synchrony is lost. (2) The song oscillator responsible for synchronous entrainment may be disturbed by heterospecific signals, which also results in a loss of synchrony with conspecific signals.

In order to account for both kinds of interference, we have chosen different experimental approaches. (1) The discrimination of males between a conspecific chirp and an artificial white noise signal was investigated in entrainment experiments in which both signals were presented in alternation or where entrainment was tested with conspecific and artificial signals of different duration. (2) Oscillator disturbances caused by various levels of rainforest noise were investigated by quantification of the variability of chirp periods of solo singing males. (3) The influence of rainforest noise on chorus synchrony was studied in playback experiments in which individual males were entrained to a periodic conspecific chirp presented at a fixed SPL of 66 dB together with rainforest noise gradually increasing in intensity.

We paralleled the behavioural approach with a neurophysiological study on the detection of conspecific chirps in a receiver using the response of an identified auditory interneuron as a marker for signal detection. The detection of conspecific signals in spike trains was performed by application of signal detection theory.

## Methods

### Insects

**Fig. 1:**
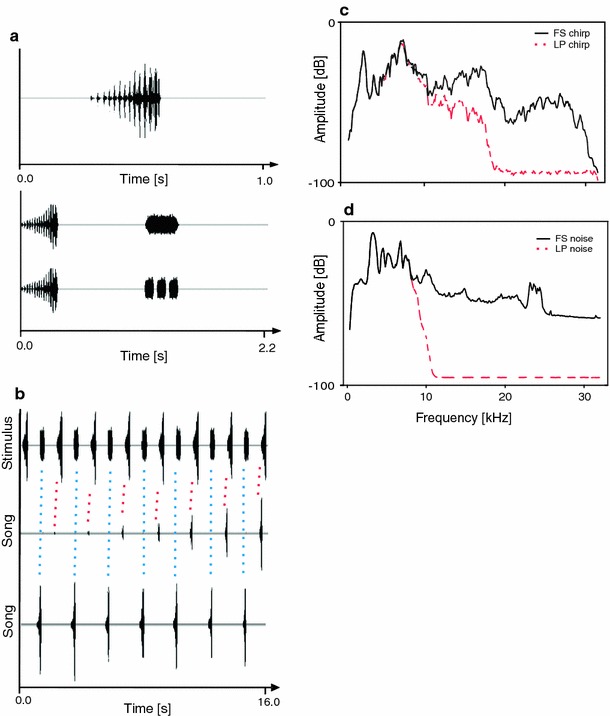
Characteristics of stimuli used in experiments. **a** (*upper panel*) Oscillogram of a conspecific chirp of *M. elongata* used in entrainment experiments. **a** (*lower panel*) Stimuli used in selective entrainment experiments consisting of a conspecific chirp alternating with either a single (*upper panel*) or triple white noise pulse. **b** Song initiation (*middle panel*) and stable entrainment (*lower panel*) of a male to a stimulus consisting of a conspecific chirp alternating with a single white noise pulse. Note the phase-locking to the chirp at the onset of entrainment (indicated by *red dotted lines*), but with the artificial pulse thereafter (indicated by *blue dotted lines*). **c** Spectrogram of a full spectrum conspecific chirp (FS) and the low-pass filtered variant (LP chirp). **d** Spectrogram of rainforest noise used as background and the low-pass filtered variant

Behavioural and neurophysiological experiments were performed with males of the katydid *M. elongata* (Orthoptera, Tettigoniidae; Mecopodinae), identical to the songs of “species S” described by Sismondo (1990). Originally these insects were collected in the field in Malaysia and later reared in crowded colonies at a temperature of 27 °C and 70 % relative humidity on a 12:12 h light:dark cycle. They were fed ad libitum with fish food, oat flakes and fresh lettuce. Males produce songs in the dark cycle consisting of regularly repeated chirps of 250–350 ms in duration (Hartbauer et al. 2005). Each chirp consists of 10–16 syllables of increasing amplitude (Fig. 1a, upper panel). The spectrum of chirps ranges from 4.5 to 95 kHz with a broadband ultrasonic component. (The spectrum shown in Fig. 1c is a low-pass filtered version of such a broadband signal). The chirp period of solo singing males is highly constant (CV ~3 %) and varies between males of a population from 1.6 to 2.4 s (mean = 2.0 s, ambient temperature 27 °C).

### Behavioural experiments

Three different behavioural experiments were conducted to discriminate between the effects on the song oscillator as a result of masking of the entraining signal or the influence of heterospecific signals at different phases of the signal period. All experiments were carried out at an ambient temperature of 27 °C: (1) The ability of individual males to discriminate conspecific chirps from white noise signals of equal energy was tested in entrainment experiments presenting both signals in alternation. (2) The susceptibility of the song oscillator to heterospecific signals within the rainforest noise was investigated by studying the variability of chirp periods of solo singing males in the presence of three SPLs of rainforest noise. (3) The influence of rainforest noise on phase-locking to a conspecific signal was studied in entrainment experiments.

### “Selective entrainment” experiment

Individual males were given a choice to synchronise with either one of two signals, a conspecific chirp or a white noise signal, presented in an alternating fashion; the sequence of signals was presented at a conspecific signal period of 2 s. After phase-locked entrainment with either signal was established for a total duration of 5 min, a gap of 1 s duration was introduced in the playback sequence in order to exchange the sequence of signal presentations. This phase-transition of the stimulus challenged males to adjust the timing of their chirps accordingly. Artificial signals and the conspecific signal in this experiment were 300 ms in duration and were calibrated to the same root mean square amplitude. Two variants of white noise signals were tested, one with a rectangular shape and a triple-pulsed signal with a single pulse duration of 80 ms interrupted by a pause of 30 ms. Both types of white noise signals were unnatural compared to the temporal pattern of syllables in conspecific chirps (Fig. 1a, lower panel). In addition, the synchrony of chirps produced right after song initiation with either signal was investigated. 15 males were tested in this experiment.

### Solo singing males

The variability of chirp periods of solo singing males was studied in the presence of rainforest noise broadcast at three different intensities (60, 65 and 70 dB SPL). Because chirp period may increase in subsequent solo song bouts generated over the whole dark period (Hartbauer et al. 2006), analysis of chirp period variability was restricted to 40 chirps produced subsequently in the first third of the first song bout (skipping the first 20 chirps in a song bout). In this segment, chirp period variability is generally low. In order to compensate for a male-specific chirp period, the coefficient of variation (CV) was calculated for 12 males.

### Entrainment under noise

The degree of phase-locked entrainment of individual males with a repetitive conspecific chirp was studied in the presence of increasing rainforest noise that was presented in two spectral versions: Either with the full spectrum of background noise as recorded in the nocturnal rainforest, or including only the sonic range below 9 kHz (LP noise, the cricket and frog fraction). The pacer signal used for entrainment consisted of a conspecific chirp repeatedly broadcast at a conspecific signal period of 2 s and a fixed intensity of 66 dB SPL. Additionally, males were entrained to a repetitive chirp lacking higher audio and ultrasonic frequencies in the presence of LP noise. This simulates the detection of a conspecific signal after transmission over some distance, which is known to attenuate higher frequencies more strongly (Römer and Lewald 1992).

**Fig. 2:**
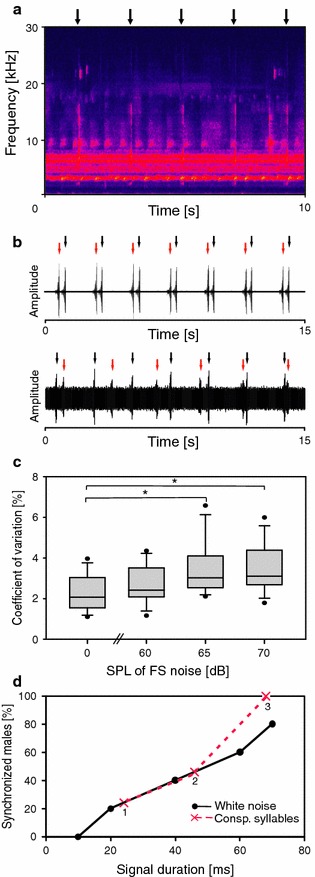
Masking potential of rainforest noise. **a** Sonogram of a stimulus situation in entrainment experiments with a playback of conspecific chirps broadcast at intervals of 2 s (*arrows*) together with a segment of rainforest noise (SNR 0 dB). **b** Examples of phase-locked entrainment of a male chirp (*black arrow*) in response to a broadcast chirp (*red arrow*) without background noise (*upper panel*) and at a SNR of −5 dB (*lower panel*). Note the loss of phase-locking in the latter case. **c** Variability of chirp period (CV) of solo singing males without background noise, and under three noise intensities. **d** Percentage of synchronised males entrained either to conspecific signals or white noise signals of different duration. Peak amplitude of signals was 66 dB SPL. *Labels* refer to the number of conspecific syllables

After males established synchronous entrainment, the SPL of rainforest noise was increased in intervals of 3 min and increments of 3 dB, beginning with 61 dB SPL and ending with 77 dB SPL. The masker was a sequence of rainforest noise (duration 200 s) presented in loop mode. A phase-locking of the pacer signal with the rainforest noise was prevented by starting the noise at randomly chosen positions. The stimulus situation with equal chirp and noise loudness (SNR = 0 dB) is shown in the sonogram of Fig. 2a. Masking with LP noise was performed at intensities from 61 to 77 dB SPL (increments of 3 dB). Synchronous entrainment was defined as the phase-locked entrainment with a pacer period of 2 s. A deviation of more than 64 ms from this period was regarded as a loss of phase-locked entrainment (see example in Fig. 2b, upper trace). This threshold corresponds to the average standard deviation of the chirp period of solo singing males determined at 27 °C (see experiment with solo singing males below). The proportion of synchronised chirps was evaluated in the last minute of a given noise playback manually for the condition of FS noise or using a custom-written macro for LP noise playbacks (Spike2, Cambridge Electronic Design, Cambridge, UK). 13 males were tested in these experiments.

In order to minimise the influence of external, uncontrolled acoustic disturbances, all behavioural experiments were performed in a temperature controlled incubator (27 °C) with the walls covered with acoustic foam. Ambient noise without playbacks was 32 dB SPL (A-weighted) which is below the hearing threshold of this species (Krüger et al. 2011). A constant distance between males and loudspeakers was maintained by caging males in a small wooden box (size 7 × 7 × 10 cm). The singing activity of males was recorded using a tie-pin microphone positioned close to the male, so that the chirp of the male could be distinguished from those of the playbacks, and detected even in playbacks of highest background noise levels. Repetitive conspecific chirps and rainforest noise was presented through separate loudspeakers positioned next to each other at a distance of 35 cm to the male.

### Sound signals

Conspecific chirps used in entrainment experiments were originally recorded at a distance of 15 cm of a solo singing male (Fig. 1a, upper panel), and thus contain a strong ultrasonic frequency component up to 90 kHz. However, spectral analysis of chirps recorded at various distances from singing males in their natural habitat revealed a strong excess attenuation of frequencies above 20 kHz (30 dB at a distance of 5 m; average of 8 males). In order to simulate this low-pass filter effect of signal transmission, the conspecific chirp used as pacer passed a steep low-pass filter with a cut-off frequency of 30 kHz, thus mimicking a signal at an inter-male distance of about 5 m (Fig. 1c, solid line). In addition, the spectral composition of a signal of a male singing at a distance of about 10 m (LP chirp) was simulated by application of a rather flat low-pass filter with a cut-off frequency of 14 kHz and a broad transition width (Fig. 1c, dashed line). Filtering was performed in CoolEdit Pro (Syntrillium Software, Phoenix, AZ, USA). Sound recordings were performed using a 1/4″ microphone (type 2540, Larson Davis, Depew, NY, USA; linear up to 45 kHz) connected to a sound level metre (CEL 414, Casella, Bedford, UK) at an ambient temperature of 27 °C. The recording was digitised by means of an A/D board (Powerlab, AD-Instruments, Spechbach, Germany) using a sampling rate of 100 kHz.

Background noise recordings performed in the natural habitat of *M. elongata* in Malaysia always included song variants of at least one of the two Mecopoda species, and thus could not be used for playbacks in entrainment experiments. Therefore, ambient rainforest noise was recorded in the understory of a moist low-land rainforest on Barro Colorado Island (Republic of Panama). This noise is rather similar in its spectral composition compared to background noise in the Malaysian rainforest (Fig. 2a; and to background noise in a lowland Indian rainforest; Balakrishnan 2005). It includes high frequency and ultrasonic signals (<30 kHz) as well as a strong frequency band below 9 kHz predominantly representing calling songs of crickets and frogs. A 200 s segment of this noise was used as background noise in the current study. A low-pass filtered rainforest noise (the ‘cricket fraction‘) termed ‘LP noise’ was obtained by application of a low-pass filter with a cut-off frequency of 9 kHz and a steep roll-off with a transition width of 600 Hz (see dashed line in Fig. 1d). Filtering was performed in Spike2 (v5.2.1, Cambridge Electronic Design, Cambridge, UK). Rainforest noise was recorded with the same microphone as conspecific chirps (see above).

The playback of sound signals was controlled in Cool Edit Pro (Syntrillium Software., Phoenix, AZ, USA) driving an external sound card (Edirol FA-101, Roland Inc., Tokyo, Japan). Sound signals were attenuated (PA-5, Tucker Davis Inc., Alachua, FL, USA) and amplified using a HIFI amplifier with a flat frequency response up to 100 kHz (NAD214, Pickering, Canada). A pair of string-tweeters (EAS-10TH400A, Technics, Kadoma, Japan) with a rather flat frequency response in the range between 200 Hz and 40 kHz was used for playbacks of conspecific chirps and rainforest noise. The same playback equipment was also used in neurophysiological experiments (see below).

In entrainment experiments, the conspecific chirp was calibrated to 66 dB SPL at the position of the singing male. According to our outdoor measurements (Hartbauer and Siegert, unpublished) this simulates a male singing at a distance of ~5–10 m. Sound calibration was performed using a 1/4″ microphone (type 2540; Larson Davis, Depew, NY, UK) connected to a sound level metre (CEL 414, Casella, Bedford, UK) operating in fast and slow reading mode for the calibration of the conspecific signal and rainforest noise, respectively. White noise sound signals were generated in the sound editing software CoolEdit Pro. The same sound calibration was used in neurophysiological experiments.

### Neurophysiology

We also used a neurophysiological approach in a total of ten individuals to study masking interference of conspecific signals with the acoustic background. Extracellular recordings of responses of a local interneuron in the prothoracic ganglion, the so-called omega neuron, were performed. For details concerning preparation and neuronal recordings see Römer et al. (2002). In short, the prothoracic ganglion was surgically exposed in a ventral side up position. An extracellular tungsten electrode was inserted close to the anterior omega tract, where the segments of the two bilaterally homologous cells cross the ganglionic midline. The preparation was placed into an anechoic chamber 35 cm distant from the loudspeakers positioned 90° laterally. Both, the conspecific signals and background noise were broadcast from the same, ipsilateral side.

Furthermore, the tuning of the omega cell to pure tone sound pulses (duration 200 ms) was determined, and intensity-response functions were obtained from nine individuals using the conspecific chirp as stimulus.

### Analysis of neuronal signal detection

A custom-written script (Spike2) was developed for the analysis of detection of chirps based on the spike rate of the omega cell. The average spike rate was calculated within a sliding time window of 30 ms, which resulted in the detection of all conspecific chirps in the control situation without noise. In order to classify bursts into hits and false alarms, the spike rate function had to cross an a priori-defined detection threshold for a minimum time interval of 15 ms, which corresponds to the duration of a single syllable. Suprathreshold events were classified either as hits when coinciding with the playback of the conspecific chirp or as false alarms, when no conspecific chirp was presented. The hit rate was calculated by dividing the number of hits by the number of presented chirps. The rate of missed chirps was obtained by subtracting the hit rate from 1. This signal detection procedure was repeated with various detection thresholds. The threshold value which yielded a maximum of hits and a lowest number of false alarms was found using a sensitivity analysis calculated after Eq. 1 (Harvey 2004)

1 $$ {\text{Sensitivity}} = \frac{{{\text{hitrate}} - {\text{false\,alarm\,rate}}}}{{1 - {\text{false alarm rate}}}}. $$

In order to study the omega cell response to rainforest noise alone, spike rates were calculated in time windows of 30 ms right after onset of the noise and in a time window of 400 ms after the firing rate reached a steady-state (Hartbauer et al. 2010).

### Statistics

Statistical tests were performed in Sigma Plot (v.12, Systat Software Inc., Chicago, IL, USA). Two groups were tested for a significant difference using a Mann–Whitney Rank Sum Test. Multi-group comparison was performed by calculating an ANOVA followed by a post hoc test.

## Results

### Selective entrainment

When males were given a choice to entrain either to a conspecific chirp or an artificial white noise stimulus, 65 % of all male chirps were generated in synchrony with the conspecific pacer, but only 35 % in synchrony with the artificial pacer, before introducing a phase-transition in the presented signals. After introducing a phase transition, 56 % of chirps were produced in synchrony with the conspecific stimulus. Results obtained with triple white noise signals were similar (Table 1). In 48 % of all experiments in which males were entrained to the conspecific chirp they did not follow a phase transition and maintained phase-locking to the conspecific pacer. In contrast, males phase-locked with the artificial pacer maintained this entrainment in only 24 % of all song bouts following a phase transition. In the remaining song bouts, males switched phase-locking to the alternative signal after introducing a phase transition. Surprisingly, during song initiation there was a much higher degree of phase-locking with a conspecific chirp compared to the artificial stimulus (see example in Fig. 1b): the first three chirps of males were produced in synchrony with a conspecific chirp in 86 % of all song bouts compared to only 14 % in synchrony with the white noise signal. This difference was significant (*p* < 0.001, *z* = 3.58, *z* Test with Yates correction). With respect to the initiation of the song there was no significant difference between single and triple-pulsed white noise signals presented in alternation with the conspecific signal (*p* > 0.05, *z* Test with Yates correction).

**Table 1:** Summary of the “selective entrainment” experiment in which males either entrained to a conspecific or an artificial pacer, both presented in alternation

Alternative signal	Before phase transition		After phase transition	
	Consp. chirp	Artificial	Consp. chirp	Artificial
Single pulse	**65.5 %** ± 42.8	**34.5 %** ± 42.8	**56.4 %** ± 44.1	**43.6 %** ± 44.1
Tripple pulse	**59.1 %** ± 43.1	**40.9 %** ± 43.1	**57.9 %** ± 44.5	**42.1 %** ± 44.5

Values represent the mean percentage (±SD) of chirps displayed in synchrony with either signal calculated across all song bouts (10–15 song bouts per male; 15 males)

When the pacer was very short (10 ms white noise pulse) males did not phase-lock at all (Fig. 2d). However, with increasing the duration of this artificial stimulus more and more males produced chirps in synchrony, so that for a duration of 70 ms about 80 % of males entrained to this stimulus with a signal period of 2 s. A similar increase in synchronisation was observed with increasing number of syllables of a conspecific chirp, and even a single syllable was sufficient to serve as a stimulus for phase-locking in some males. With a stimulus consisting of only three syllables, we observed 100 % synchronous entrainment in all males (*N* = 9).

### Solo singing males

When males were allowed to sing in isolation without background noise, the coefficient of variation of the chirp period was rather low with 2.1 % (mean CV of 12 males), rather similar to the value determined in a previous study (Hartbauer et al. 2006). The CV slightly increased to 2.4 % during playbacks with background noise presented at 60 dB SPL (Fig. 2c). A further increase of the noise intensity resulted in a significant increase of CV to 3.0 and 3.1 % for 65 dB and 70 dB SPL, respectively (*p* < 0.05, One-way ANOVA on ranks).

### Entrainment under noise

**Fig. 3:**
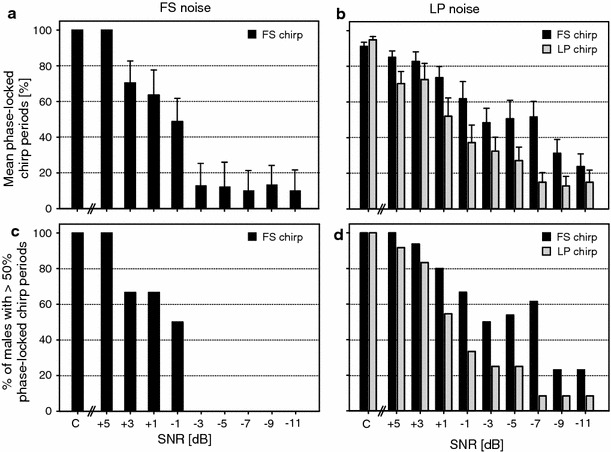
Influence of rainforest noise on the phase-locked entrainment of *M. elongata* males. **a** Average proportion of chirps produced at a similar period compared to a conspecific stimulus used as pacer in the control (no noise) and under various levels of background noise. **c** Percentage of males with more than 50 % of chirp periods similar to the pacer period (2 s). **b**, **d** The same as in **a** and **c** but using LP noise as masker. *Grey columns* represent results using a LP chirp as pacer. *Error bars* indicate SEM

In the absence of noise (see example in Fig. 2b, upper trace) and under low noise conditions (60 dB SPL; SNR +6 dB), phase-locked entrainment with a conspecific chirp as pacer was perfect (Fig. 3a). With increasing background noise levels and thus decreasing SNR the percentage of chirp periods matching the entrainment period quickly dropped (see example in Fig. 2b, lower trace). With a SNR of +1 dB, 65 % of males still produced 50 % of their chirps at a period of 2 ± 0.064 s (Fig. 3c), and the mean percentage of chirp period match was 62 % (Fig. 3a). A noise intensity that exceeded chirp intensity by 3 dB and higher reduced phase-locked entrainment to values of about 10 % (see for example Fig. 2b; lower panel). The influence of the “cricket fraction” of rainforest noise (LP noise) on phase-locked entrainment was similar (Fig. 3b, d). However, the proportion of period matching decreased more gradually with increasing noise intensity (Fig. 3b). A SNR of +1 dB allowed 80 % of males to produce 50 % of their chirps at periods similar to the pacer period (Fig. 3d). When males were entrained to a conspecific stimulus with reduced frequency content at higher audio and ultrasonic frequencies (LP chirp) the percentage of period matching at medium and higher noise intensities was reduced.

### Neurophysiology

**Fig. 4:**
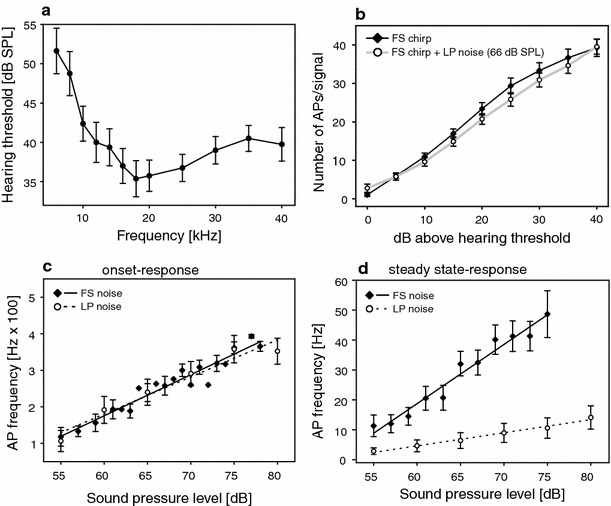
Neuronal response to conspecific signals and rainforest noise presented at various intensities. **a** Average tuning curve of the omega neuron (8 preparations). **b** Intensity-response functions using a conspecific chirp as stimulus. *Open symbols* response to conspecific chirps in the presence of LP noise (average of 9 preparations). **c** Onset response of the omega neuron to full spectrum rainforest noise and LP noise increasing in intensity. **d** Steady-state response of the omega neuron in playbacks of different noise variants presented at increasing SPL (**c**, **d** data from 8 preparations). *Error bars* indicate SD

Omega neurons of *M. elongata* are most sensitive to a broad frequency range above 10 kHz (Fig. 4a), with a moderate roll-off at lower frequencies. The intensity-response function of the neuron for the conspecific signal as stimulus was linear up to 40 dB above threshold (Fig. 4b), and the response was almost unaffected by LP rainforest noise (*p* > 0.05, ANOVA on ranks, Fig. 4b).

**Fig. 5:**
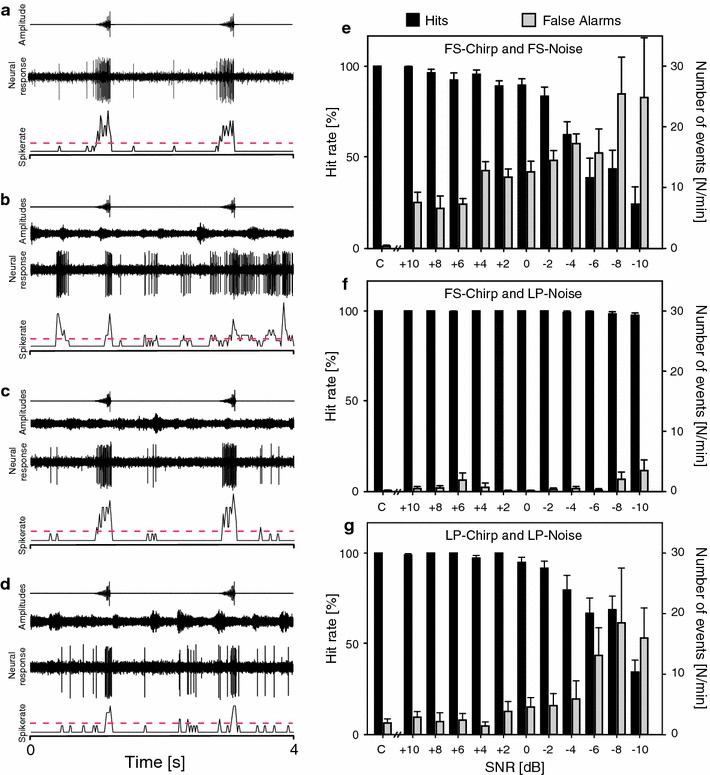
Omega cell response to conspecific chirps under various background noise conditions. Examples of omega cell responses to conspecific chirps **a** without background noise, **b** under the full spectrum noise, **c** under LP noise, and **d** of the LP-filtered chirp under LP-filtered noise. For all responses, the spike rates are plotted below the recording examples with a threshold for signal discrimination (for further explanation see M&M). Hits and false alarm rates calculated from spike rate functions under **e** the full spectrum noise, **f** the LP-filtered noise, and **g** the LP-filtered noise with a LP-filtered chirp. Data from 10 preparations. *Error bars* indicate SD

The response of the neuron to the different variants of background noise is shown in Fig. 4c (onset response) and Fig. 4d (steady-state-response). The onset of rainforest noise caused an intensity-dependent increase of the response of the neuron determined within the first 30 ms following stimulus onset (Fig. 4c). Interestingly, no difference was found in the onset response to FS and LP rainforest noise, although steady-state spike rates of both noise variants differed strongly, since increasing intensity of FS noise caused a stronger increase of the steady-state response compared to LP noise (Fig. 4d). The omega cell responded to the presentation of conspecific chirps by encoding the amplitude increase of syllables in spike count (Fig. 5a).

Background noise affected the neuronal representation of conspecific chirps depending on the spectral composition of both the noise and signal. Figure 5 shows representative examples of the spiking activity of the omega neuron in response to the FS signal under FS and LP noise (Fig. 5b, c, respectively), and the LP signal under LP noise (Fig. 5d). The major effect of the FS background noise is an increase in the number of bursts elicited by high-frequency or ultrasonic events in the background, which either add to bursts elicited by the conspecific signal or interfere with them in time (compare Fig. 5a with 5b). Such a masking effect did not occur with LP noise because the acoustic energy in the background relevant for masking has been filtered out (Fig. 5c). Similarly, a LP-filtered conspecific chirp elicited a shorter burst in the neuron compared to FS chirp (compare responses to chirps in Fig. 5c with d).

These masking effects of rainforest noise were further quantified by calculating hit and false alarm rates based on suprathreshold bursts in the omega cell response (Fig. 5e–g). FS background noise resulted in two effects for signal detection: a decrease of the average hit rate, and simultaneously an increase in false alarm rates, which gradually increased with increasing background noise intensity (Fig. 5e). Although the hit rate was still high at a SNR of 0 dB, false alarm rate increased to an average of 15 events in a time period of 60 s, which corresponds to one false alarm in every second chirp period. By contrast, LP noise did not interfere with the detection of conspecific chirps at all, even at SNRs as low as −10 dB (Fig. 5f). In response to LP chirps under LP noise the hit rate was almost unaffected up to a SNR of 0 dB (Fig. 5 g), but then gradually decreased to values well below 50 % at a SNR of −10 dB, and concomitantly the false alarm rate increased to values of about 15–20 events/min.

## Discussion

The high diversity of acoustic signallers in the nocturnal rainforest produces a complex background in the audio and ultrasonic range with the potential of masking interference with the broadband advertisement signals of *M. elongata*. The hearing organ of *M. elongata* (Krüger et al. 2011) and all katydids studied so far covers a large frequency range from <5 kHz up to the high ultrasonics (Rheinlaender et al. 1972; Römer et al. 1989; Stumpner and Molina 2006). Thus, receivers should be sensitive to both conspecific signals and background noise. In our behavioural paradigm, we took advantage of the fact that males readily synchronise with the chirps of one or more males within earshot (Sismondo 1990), so that the masking effect of background noise can be quantified from the degree of synchrony of chirps with a song model in entrainment experiments. Results in these behavioural experiments revealed that synchronous entrainment is completely unaffected by background noise at SNR of +5 dB (100 % synchronisation), and even at SNRs of −1 dB period matching occurred in about 50 % of events (Fig. 3a). Thus, males will be able to maintain chorus synchrony in the presence of realistic levels of background noise. Considering the complexity of the masker this tolerance is remarkable and will be discussed below.

### Recognition of conspecific signals

Results obtained in “selective entrainment” experiments suggest that males appear to discriminate conspecific signals from artificial ones during song initiation (Fig. 1b), since they were more likely to synchronise with chirps compared to simple white noise bursts of the same amplitude. However, after the establishment of a synchronous entrainment, a phase shift that re-establishes synchrony with a conspecific signal was only observed in 50 % of song bouts. This suggests that males may simply rely on the duration of the conspecific signal period for maintaining synchronous entrainment with a pacer. In this case males detect periodic signals rather than recognising certain signal components like the specific amplitude modulation of chirps or the syllable period. This view is corroborated by results of entrainment experiments with artificial signals and chirps differing in duration (Fig. 2d), where males synchronised with a signal consisting of only three syllables (duration about 70 ms), but even an unmodulated white noise sound pulse of a similar duration yielded about 80 % synchrony.

The lack of discrimination based on the species-specific chirp structure appears to be adaptive considering the strong overlap of chirps in a chorus situation, which inevitably masks the syllable pattern of individual chirps. So far, we do not know whether females in this species fail to discriminate such signal features as well, and experiments using female phonotaxis as a behavioural paradigm have just started. However, previous studies on female preferences indicate that the relative call timing of males is more important for mate choice compared to signal parameters suffering from masking interference in a chorus situation (Fertschai et al. 2007). Therefore, the maintenance of chorus synchrony in a noisy environment is different from a situation in which receivers discriminate signals by means of assessing certain call parameters like amplitude modulation, carrier frequency, spectral composition or frequency modulation (Kriegbaum 1989; von Helversen and von Helversen 1997; Lengagne et al. 2001; Richardson and Lengagne 2010). Behavioural studies in birds (Lohr et al. 2003) and frogs (Wollerman and Wiley 2002) as well as psychophysics (Swets et al. 1978) revealed a higher threshold for signal discrimination compared to signal detection (Wiley 2006). Apparently, signal detection constitutes a less challenging task compared to discriminating between different signals. Therefore, we interpret the high noise tolerance in *M. elongata* as the outcome of a robust detection of signals reoccurring at a conspecific period of about 2 s.

### Perturbations of the song oscillator

Although such a strategy enables chorus synchrony, heterospecific signals within the acoustic background may have the potential to disturb the song oscillator that responds with a phase shift to disturbing signals which are out of phase with the oscillator rhythm. The degree of the resulting phase shift depends on the intensity and the phase of perturbation (see corresponding phase response curves in Sismondo 1990; Hartbauer et al. 2005). Surprisingly, when solo singing males were exposed to varying levels of background noise the song oscillator of *M. elongata* was only marginally affected. We found a significant difference in the variation of the chirp period under noise at levels of 65 and 70 dB SPL compared to the noiseless condition (Fig. 2c), but a coefficient of variation of 3 % corresponds to a chirp-to-chirp period variability of only 60 ms, which is just below the threshold for classifying chirp periods as asynchronous. Although such a precision cannot compete with most regular biological oscillator known from the electric organ discharge of weakly electric fish (Moortgat et al. 2000), the main reason for the observed decrease of synchronisation in the presence of noise at SNRs of +3 dB and less is the consequence of masking interference of conspecific signals with signals in the acoustic background lacking a species-specific signal period. This likely interferes with the detection of periodic signals. It seems that for the maintenance of chorus synchrony in the presence of moderate levels of noise, males are able to detect a signal presented with a conspecific period by simultaneously ignoring heterospecific signals lacking this species-specific signal period.

The nocturnal background noise is characterised by a strong audio component below 10 kHz, and high audio and ultrasonic components (Figs. 1d, 2a). The results of entrainment experiments under LP noise indicate a high tolerance for this frequency band compared to higher frequencies: For example, at a SNR of −7 dB males still produced about 50 % of chirps at periods similar to the pacer period, whereas with the FS noise this value occurred at a SNR of −1 dB. This is due to the fact that acoustic events in the background that might interfere with synchronous entrainment with a pacer have their main energy at higher frequencies, which is well reflected in the neurophysiological results.

### Neuronal representation of chirps under noise

As a monitor for the representation of conspecific signals and noisy events in the CNS of receivers, we studied the response of the so-called omega neuron, a local interneuron in the prothoracic ganglion of katydid and cricket species. The reason for choosing this neuron was not its potential function in a neuronal network for species-specific signal detection, but the fact that its activity reflects the input of almost all sensory receptor neurons in the ear (except those at the low-frequency end of the hearing range; Römer 1985; Stumpner and Molina 2006). As the neuron fires tonically in response to all kinds of transient stimuli within its range of frequency tuning (Fig. 4a), it is ideally suited to examine whether information about conspecific signals is represented at a very early level within the auditory pathway even under realistic environmental noise levels.

Responses to conspecific chirps are represented in the receiver as strong bursts of action potentials, but noisy events in the background may induce similar responses (Fig. 5b), so that the incidence of false alarms increases with decreasing SNRs, while the rate of hits decreases (Fig. 5e). Thus, these false alarms are likely responsible for the breakdown of synchronous singing documented with the full spectrum noise (Fig. 3a). Since in the LP-filtered noise the number of noisy events for such interference is strongly reduced, this would explain the reduced decrease in phase-locking, although the behavioural data do not match quantitatively with the almost complete lack of false alarms when using the LP noise as background (Fig. 5f). Two factors may account for this discrepancy. First, as mentioned above, the omega cell is a local interneuron in the prothoracic ganglion and does not provide higher brain areas with information about acoustic signals. Apparently, the behaviour of males is more sensitive to events in the LP noise than the false alarm rate of this neuron would suggest. Therefore, the neural network driving song synchrony must have a frequency tuning somewhat more sensitive towards lower frequencies, which may explain the strong frequency band in conspecific signals around 8 kHz (Fig. 1c). As a second possibility, the discrimination threshold used for detecting hits and false alarms may not represent the real threshold in the network responsible for entrainment, so that false alarms might be underrepresented in the LP noise background regime (Fig. 5f). However, although the actual value of such a threshold (see M&M for the algorithm) might be disputable, the chosen algorithm ignored all spontaneous activity without burst character, but still allowed the detection of the reduced responses to chirps where high frequency components have been filtered out (Fig. 5g).

Under natural conditions the transmission of the broadband frequency spectrum of the conspecific chirp will result in a frequency-dependent decrease in intensity, so that at some distance from a male the signal will include no power in the ultrasonic range and reduced power at high frequency components (the simulated LP chirp). LP noise interfered with phase-locked entrainment much stronger when the pacer was a LP chirp. In this case, playbacks resulted in a lower hit rate and a higher rate of false alarms with decreasing SNRs (Fig. 5g). Thus, both behavioural and neurophysiological results suggest that synchronous interactions between males of *M. elongata* are more likely at intermediate nearest neighbour distances in the range of 5–10 m, a value that was also found in an Indian species of the *M. elongata* group (Nityananda and Balakrishnan 2008).

In an attempt to demonstrate that the ultimately highly important information about the presence of a predator is still represented in the afferent sensory pathway of katydids, and can be read out from the central nervous system despite considerable ambient background noise, Hartbauer et al. (2010) developed an algorithm which used the specific timing of action potentials within bursts, and the repetition of bursts in omega cell responses. Their results allowed successful detection of predator events (bat echolocation calls) in spike trains even under strong background noise. Similarly, for the present study one might argue that more sophisticated analysis of spike timing in burst responses may allow discriminating bursts in response to conspecific chirps from those in response to background noise. However, the calling behaviour of males in the presence of conspecific and artificial entrainment signals does not indicate that males make use of this information.

In summary, a high robustness of the song oscillator with respect to heterospecific signals in the background favours the production of chirps with only little deviation from the conspecific signal period. This facilitates chorus synchrony by providing other males a species-specific pacer that can be detected in the presence of realistic noise levels. Our results suggest that males pay attention to signals repeated with a species-specific signal period while ignoring other signals lacking this periodicity. From the viewpoint of female receivers such a noise tolerance is also important because a lack of noise tolerance would disrupt a consistent leader–follower relationship as a cue for mate choice (Fertschai et al. 2007).

## Abbreviations

SPL: Sound pressure level
LP: Low-pass filtered
FS: Full spectrum
SNR: Signal-to-noise ratio
